# Flight and Interaction Control of an Innovative Ducted Fan Aerial Manipulator

**DOI:** 10.3390/s20113019

**Published:** 2020-05-26

**Authors:** Yibo Zhang, Bin Xu, Changle Xiang, Wei Fan, Tianfu Ai

**Affiliations:** 1 School of Mechanical Engineering, Beijing Institute of Technology, Beijing 100081, China; yibo1993@gmail.com (Y.Z.); xiangcl@bit.edu.cn (C.X.); aitianfu1994@gmail.com (T.A.); 2 Chongqing Innovation Center, Beijing Institute of Technology, Chongqing 401147, China; 3 School of Automation, Beijing Institute of Technology, Beijing 100081, China; fanweixx@bit.edu.cn

**Keywords:** aerial manipulator, ducted fan, adaptive control, impedance control

## Abstract

An innovative aerial manipulator with ducted fans is proposed to achieve side-on aerial manipulation tasks in a confined environment, such as canopy sampling in dense forests. The dynamic model of the novel design is studied, and on this basis a composite controller is proposed to address the challenges of arm extension and physical interaction during the manipulation process. An adaptive controller is proposed for the aerial platform to achieve good stability and tracking performance under the manipulator motion, and an impedance controller is designed for the manipulator to ensure compliance and stability during physical contact. The experimental tests validate the effectiveness of the proposed prototype structure and controller design.

## 1. Introduction

The application of an unmanned aerial vehicle (UAV) has become more and more extensive. The information interaction between a UAV and the environment (such as aerial photography [1], monitoring [2], target tracking [3], etc.) has been unable to meet the increasing requirements. Otherwise, with the development of small vertical takeoff and landing (VTOL) UAV, the aerial physical interaction (aerial manipulation) has begun to receive attention in the past decade, and the AIRobots project, AEROWORKS project, and ARCAS project in Europe are the most representative works. AIRobots aims to develop a new generation of aerial service robots capable of contacting the environment actively and safely [4]; AEROWORKS aims to design collaborative aerial robotic workers for infrastructure inspection and maintenance [5]; ARCAS develops unmanned flight systems for assembly and structure construction [6]. Through the combination of a UAV and robot arm, the aerial manipulator system can expand the operation ability of a ground robot into the air. Using an aerial manipulator instead of the workforce to complete tasks can significantly improve efficiency, reduce costs, and ensure human safety. For example, the aerial manipulator can quickly complete sample collection in wide-area space [7], can replace the workforce for infrastructure maintenance and repair [8], and can participate in firefighting in dangerous environment [9].

The configuration of the aerial manipulator commonly consists of a flight platform and an operation manipulator. In terms of structure composition, most aerial manipulators use helicopter or multirotor as flight platforms because of their simple structure and adequate research. The Chinese Academy of Sciences used a quadrotor to slung loads by a cable [10]. Yale University used a helicopter combined with a single-degree-of-freedom (DOF) gripper to achieve compliant grasping of the object under the platform [11]. Drexel University also used helicopter as the platform, expanding the 1-DOF gripper to a 7-DOF robot arm to achieve arbitrary gesture of the end effector, to complete the peg-in-hole test [12]. For the purpose of bridge detection, University of Seville combined a multirotor with a 5-DOF manipulator to achieve contact control of the plane above the aircraft [13]. In addition, University of Seville has also proposed a dual manipulator system to facilitate the collaborative grasping of a bar [14]. However, the actual working environment of aerial robots is usually complex and confined. In such an environment, the above-mentioned open rotor platform is unsafe, with low trafficability, and cannot interact with the environment closely. Besides, in order to avoid the rotor disc, the robot arm can only approach the target from above [11] or below [13], which greatly limits the application ranges. For realizing side-on manipulation, Johns Hopkins University designed a very long arm, which can extend out from the rotors [15], but the long arm brings great changes in the center of gravity and moment of inertia of the system, posing great challenges to the system stability. The University of Auckland installed a manipulator on the side edge of the rotorcraft frame [16]. Although the side branches sampling was realized, the offset of the system’s center of gravity significantly affected the control accuracy. Compared with helicopter and multirotor, ducted fan has greater thrust and payload with a smaller structure size, and the duct also makes it safer for flying and interaction in the confined environment [17]. University of Bologna proposed a single-ducted fan aircraft and completed physical contact experiment [18], but due to the payload and controllability limitations, their aircraft cannot carry the robot arm.

The aerial manipulation process can generally be divided into four stages [19]: takeoff and free flight, approaching and arm extension, contact and manipulation, retraction, and departure. In terms of system control, compared with traditional UAVs, there are two main challenges in aerial manipulation control. First, in the approaching stage, the aerial platform will be severely disturbed under the manipulator motion, which is far beyond ordinary disturbances such as wind gusts, thus both the stability and tracking accuracy are greatly challenged. Second, in the contact stage, the transient force caused by the physical interaction between the end effector and the environment poses a great challenge to the stability of the system, and the contact compliance must be ensured.

As to the first challenge, Yale University ignored the impact of the manipulator on the helicopter platform by using a lightweight 1-DOF gripper [11]. A linear LQR controller is designed by Chinese Academy of Sciences to ensure the robustness of the aircraft, and the disturbance boundary is guaranteed by limiting the motion range of the manipulator [20]. Utah State University compensated the deviation of center of mass caused by the manipulator motion through variable parameter integral back-stepping (VPIB) controller, but the inertia force and moment of the manipulator are not considered [21]. Harbin Institute of Technology (Shenzhen) guaranteed the positioning performance of the gripper through visual servo compensation while the aerial platform is disturbed by the manipulator motion [22,23]. As to the second challenge, Yale University guaranteed stability in the grasping process by using an under-actuated passive compliant gripper [11]. ETH Zurich achieved compliance with the environment of a quadrotor (without the arm) under external force through admittance control. However, correspondingly, compliance means its positioning accuracy under disturbance cannot be guaranteed [24]. Additionally, ETH Zurich achieved stable physical contact between the rotorcraft (by a bracket) and the wall through model predictive control method [25]. Seoul National University applied the passive decomposition method to realize the contact control of the aerial manipulator through the force/motion hybrid controller [26]. However, both of them involve contact force detection and control law switching, which is complex and not easy to practice.

Towards the discussion above, the contribution of this work has two parts. First, an aerial manipulator system composed of tandem ducted fans is introduced. Compared with traditional UAV platforms, such as helicopters and multirotors, the proposed aerial manipulator can interact with the complex confined environment more closely and operate from side-on easily with a smaller range of joint motion. Second, considering both the arm motion and end effector interaction, a composite controller of the aerial manipulator is proposed. An adaptive controller is proposed for the aerial platform to achieve good stability and positioning performance under the manipulator motion. Both the static and dynamic forces of the arm acting on the platform are estimated and compensated by the proposed controller. Additionally, an impedance controller is used for the manipulator to ensure compliance and stability during physical interaction. The designed controller is easy to implement without measuring contact forces or switching control laws during interaction.

In the following, Section 2 introduces the aerial manipulator prototype, and the system model is established. Then the composite controller design is detailed in Section 3, and the experimental verification is presented and analyzed in Section 4. Finally, some conclusions are drawn in Section 5.

## 2. Modeling of the Aerial Manipulator

### 2.1. System Introduction

**Figure 1:**
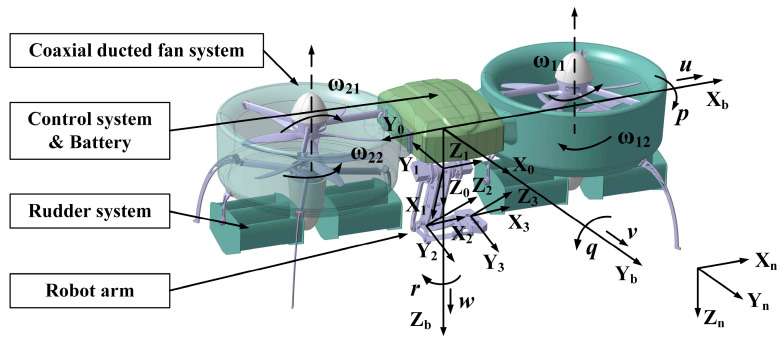
The configuration and coordinate frame of the system.

Figure 1 shows the proposed tandem ducted fan aerial manipulator, which is mainly composed of four parts: two ducted coaxial rotor systems, two sets of rudder systems set below the ducts, a 3-DOF manipulator, and the control and power system. The weight of the proposed robot is 5.5 kg, and the maximum grasping weight is 0.5 kg. The key parameters of the aerial vehicle prototype are given in Table 1. The difference of rotor speeds between the front and rear ducted fans causes the difference of thrust, which generates pitching torque to control the pitch channel. The deflection angle of the rudder causes rolling torque to control the roll channel. The difference of rotor speeds between the upper and lower rotors in each duct causes the difference of reaction torque, which is to control the yaw channel. The manipulator is set on the body center of the vehicle platform, and its 3 DOF (one lumbar joint, one shoulder joint, and one elbow joint) makes it possible for the end effector to reach any position in 3D space. Since our application purpose only concerns about the position of the end effector, the posture of it is not considered.

**Figure 2:**
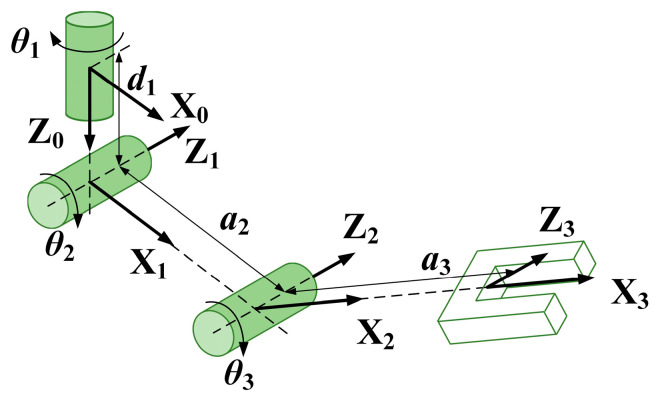
The D–H frame of the manipulator.

The proposed aerial manipulator is a multi-body system with four interconnected rigid bodies, namely, the aerial vehicle body, and the three links of the manipulator. The coordinate system is also described in Figure 1, let Σ*_n_* -{X*_n_*, Y*_n_*, Z*_n_*} be the earth-fixed coordinate frame, Σ*_b_* -{X*_b_*, Y*_b_*, Z*_b_*} be the vehicle body-fixed coordinate frame at the center of mass of the vehicle, Σ**_0_ −{X**_0_, Y**_0_, Z**_0_} be the manipulator base-fixed coordinate frame, and Σ*_i_* −{X*_i_*, Y*_i_*, Z*_i_*} (*i* = 1, 2, 3) be the coordinate system of each link of the arm. The coordinate definition and the parameter description of the manipulator use the standard D–H (Denavit–Hartenberg) rules [27], as shown in Figure 1. Additionally, Figure 2 illustrates the D–H frame with a more intuitive way, and the D–H parameters are given in Table 2. Notice that Σ**_0_ coincides with the origin of Σ*_b_*, only rotated 90 degrees around the Z*_b_* axis.

### 2.2. Aerial Vehicle

#### 2.2.1. Kinematic Model

Define the position and Euler angle vectors of the aerial platform in the inertial (earth-fixed) coordinate system as ***p****_b_* = [*x_b_ y_b_ z_b_*]^T^ and ***Φ**_b_* = [*φ θ ψ*]^T^, and the linear velocity vector and attitude angular rate vector in body-fixed coordinate system as *^b^**v**_b_* = [*u_b_ v_b_ w_b_*]^T^ and *^b^**ω**_b_* = [*p q r*]^T^, where the superscript *b* denotes that the variable is expressed in the body-fixed frame. The kinematic model of the aerial vehicle can be derived as

(1) vbb=p˙bb=RTbnp˙bωbb=RTbnωb=RTbnTbΦ˙b=QbΦ˙b

where ${}_{b}^{n}$***R*** denotes the rotation matrix of body-fixed frame Σ*_b_* with respect to the earth-fixed frame Σ*_n_*, ***T****_b_* is the transformation mapping matrix between the time derivative of Euler angle and the angular rate in the inertial frame, and accordingly ***Q****_b_* is the transformation matrix when it refers to the body-fixed frame, which are given as

(2) Rbn=[cosψcosθcosψsinθsinφ−sinψcosφcosψsinθcosφ+sinψsinφsinψcosθsinψsinθsinφ+cosψcosφsinψsinθcosφ−cosψsinφ−sinθcosθsinφcosθcosφ]Qb=[10−sinθ0cosφcosθsinφ0−sinφcosθcosφ]

#### 2.2.2. Dynamic Model

The aerial vehicle platform’s dynamic model then can be derived by the Newton–Euler formulation [27] as

(3) Fuavb+RTbnmbg−farmb=mb(v˙bb+ωbb×vbb)Muavb−narmb=Ibω˙bb+ωbb×Ibωbb

where *m_b_* is the mass of the platform, ***I****_b_* is the inertia matrix, and *^b^**F**_uav_* and *^b^**M**_uav_* are the platform’s resultant force vector and resultant moment vector with respect to the body-fixed frame respectively. The most important part of the resultant force vector *^b^**F**_uav_* is the thrust of the coaxial ducted fans, which provides the lift of the vehicle. Notice that in addition to the rotors, the duct itself can generate additional thrust during its aerodynamic structure [28], which is beneficial to the effective operation payload. The resultant force also includes the aerodynamic drag of the ducted fans, the aerodynamic force of the rudders, and the fuselage resistance. The resultant moment vector *^b^**M**_uav_* includes the torque generated by the rudder forces, the torque generated by the differential ducted fan thrusts, the reaction torque of the rotors, providing the control torques of roll, pitch, and yaw channel respectively. Additionally, also, the resultant moment includes the aerodynamic moment and gyro moment of the rotors. The dynamic and aerodynamic characteristic analyses of the ducted fan system are studied in our previous works [29,30], and will not be detailed here. *^b^**f**_arm_* and *^b^**n**_arm_* are the force and moment exerted on the vehicle platform by the manipulator arm, which will be introduced in the next part.

The dynamic mechanism model in Equation (3) of the aerial platform is a high order, nonlinear, multi-input multi-output (MIMO) system with strong coupling. Since the aerial platform works in hovering or near-hovering condition in the aerial manipulation scenarios, to facilitate the controller design process in Section 3.2, a linear state space model is identified at the hovering condition using the frequency domain identification method. Following the identification procedure in [31], and let ***η****_b_* = [*^b^*$v_{b}^{T}$ *^b^*$\omega_{b}^{T}$ $\Phi_{b}^{T}$]^T^ be the state variables, referring to the linear velocities, angular rates and Euler angles of the aerial vehicle; let ***ς****_b_* = [*^b^*$v_{b}^{T}$ *r*]^T^ be the output variables, referring to the linear velocities in three directions and yaw angular rate. Then the simplified state space model of the aerial vehicle can be described as

(4) $$\begin{array}{l} {\dot{\eta }}_{b}\left(t\right)=A_{b}\eta_{b}\left(t\right)+B_{b}u_{b}\left(t\right)+\mu_{m}\left(t\right)\\ \varsigma_{b}\left(t\right)=C_{b}\eta_{b}\left(t\right) \end{array}$$

where ***u****_b_* = [*u_alt_ u_rol_ u_pit_ u_yaw_*]^T^ is the control input vector, mapping to the rotor speeds and rudder angles as introduced in 2.1, and is normalized to [−1, 1]. The subscripts of *u* denote the altitude, roll, pitch, and yaw channel respectively. ***μ****_m_* is the force and moment vector of the manipulator acting on the vehicle body. ***A****_b_* and ***B****_b_* are the state matrix and control matrix respectively, ***C****_b_* is the output selection matrix. The identified results of ***A****_b_* and ***B****_b_* are

(5) $$A_{b}=\left(\begin{array}{ccccccccc} -0.0876 & 0 & 0 & 0 & 0 & 0 & 0 & -9.8010 & 0\\ 0 & -0.0876 & 0 & 0 & 0 & 0 & 9.8010 & 0 & 0\\ -0.1178 & -0.1172 & -1.0415 & -0.0012 & -0.0209 & -0.0509 & 0 & 0 & 0\\ 0 & -0.6801 & 0 & 0 & 0 & 0 & 0 & 0 & 0\\ 0.0940 & 0 & 0 & 0 & -1.0699 & 0.0132 & 0 & 0 & 0\\ 0 & 0 & 0 & 0 & -0.0122 & 0 & 0 & 0 & 0\\ 0 & 0 & 0 & 1 & 0 & 0 & 0 & 0 & 0\\ 0 & 0 & 0 & 0 & 1 & 0 & 0 & 0 & 0\\ 0 & 0 & 0 & 0 & 0 & 1 & 0 & 0 & 0 \end{array}\right),\;B_{b}=\left(\begin{array}{cccc} 0 & 0 & 0 & 0\\ 0 & 0 & 0 & 0\\ -20.9312 & 0 & 0.0547 & -0.0015\\ 0 & 8.3494 & 0 & 0.0260\\ 0 & -0.0025 & 10.0876 & -0.0281\\ 0 & 0.0016 & 0.1337 & 3.3981\\ 0 & 0 & 0 & 0\\ 0 & 0 & 0 & 0\\ 0 & 0 & 0 & 0 \end{array}\right)$$

### 2.3. Manipulator

#### 2.3.1. Kinematic Model

Define the *q_i_* (*i* = 1, 2, 3) to be the joint angle of the manipulator and ***v****_i_* and ***ω****_i_* are the velocity vector and angular rate vector of the origin of link *i*. The superscript *i* denotes that the variable is respect to the coordinate frame Σ*_i_*, and specially, 0 denotes the manipulator base-fixed coordinate frame, which can be considered as link 0. The kinematics of the manipulator can be derived iteratively as

(6) v00=Rbb0vbvii=Ri−1i(vi−1i−1+ωi−1i−1×pii−1)ω00=Rb0ωbbωii=Ri−1i−1iωi−1+q˙iezi

where *^b^**v**_b_* and *^b^**ω**_b_* are the linear velocity and attitude angular rate of the vehicle platform in the body-fixed coordinate frame, calculated by Equation (1). ${}_{-1}^{i}$***R*** is the rotation matrix between the coordinate frame of link *i* and link *i*−1, and specially, ${}_{b}^{0}$***R*** is the rotation matrix between the manipulator base-fixed coordinate frame and the vehicle body-fixed coordinate frame.*^i^*^−1^***p****_i_* is the position vector of the origin of link *i* in the coordinate frame Σ*_i_*_−1_. ***e***_z*i*_ denotes the projection along Z axis of link *i*.

Let Σ*_e_* be the end effector-fixed coordinate frame, which coincides with the origin of Σ_3_ with a rotation transformation ${}_{3}^{e}$***R***. Rearrange Equation (6) with a compact form, the velocity vector of the end effector with respect to the body-fixed frame can be described as

(7) p˙eb=Jeb(q)q˙

where ***p****_e_* = [*x_e_ y_e_ z_e_*]^T^ is the position vector of the end effector, ***q*** = [*q*_1_ *q*_2_ *q*_3_]^T^ is the joint angle vector, ***J****_eb_*(***q***) is called manipulator Jacobian matrix with respect to the vehicle frame. Then with the combination of Equation (1), the end effector velocity in Equation (7) can be expressed in the inertial frame as

(8) $${\dot{p}}_{e}=J_{b}\left(q,\;\Phi_{b}\right){\dot{\xi }}_{b}+J_{e}\left(q,\;\Phi_{b}\right)\dot{q}$$

where ***ξ****_b_* = [$p_{b}^{T}$ $\Phi_{b}^{T}$]^T^ is the position and attitude state vector of the vehicle platform, ***J****_b_*(***q***, ***Φ****_b_*) and ***J****_e_*(***q***, ***Φ****_b_*) are the vehicle and manipulator Jacobian matrix with respect to the inertial frame respectively, which are functions of joint angle ***q*** and vehicle attitude ***Φ****_b_*, and ***J****_e_*(***q***, ***Φ****_b_*) = ${}_{b}^{n}$***RJ**_eb_*(***q***).

#### 2.3.2. Dynamic Model

The dynamic model of the manipulator can then be derived by the recursive Newton–Euler (RNE) method [27]. First, based on Equation (6), the resultant force ***F****_i_* and moment ***M****_i_* of each link *i* of the manipulator can be calculated outward iteratively from the aerial vehicle to the robot end effector as

(9) Fii=miv˙CiiMii=Iiω˙ii+ωii×Iiωii

where *m_i_* and ***I****_i_* are the mass and inertia matrix of link *i*. The linear acceleration of the mass center of link i and the angular acceleration of link i are given by

(10) ω˙ii=Ri−1iω˙i−1i−1+Ri−1iωi−1i−1×q˙iezi+q¨ieziv˙ii=Ri−1i(v˙i−1i−1+ω˙i−1i−1×pii−1+ωi−1i−1×(ωi−1i−1×pii−1))v˙Cii=v˙ii+ω˙ii×pCii+ωii×(ωii×pCii)

where *^i^**p**_Ci_* is the position vector of the mass center of link *i* with respect to the coordinate frame Σ*_i_*. Then the joint torque *τ_i_* of each link *i* can be calculated inward iteratively from the robot end effector to the aerial vehicle as

(11) fii=Fii+Ri+1ifi+1i+1−RTinmignii=Mii+Ri+1ini+1i+1+pi+1i×Ri+1ifi+1i+1+pCii×(Fii−RTinmig)τi=(nii)Tezi

where *^i^**f**_i_* and *^i^**n**_i_* are the force and moment exerted on link *i* by link *i*−1 with respect to the coordinate frame Σ*_i_*. Notice that the variables with subscript *i* + 1 when *i* = 3 refers to the force and moment exerted on the end effector from the environment. Additionally, specially, the force and moment exerted on the aerial platform by the manipulator arm, namely, *^b^**f**_arm_* and *^b^**n**_arm_* as mentioned in Section 2.2, can be calculated as

(12) farmb=R0bf00narmb=R0bn00

Rearranged Equations (9)–(11) the dynamic formula of the manipulator can be expressed in body-fixed frame with a compact form as

(13) $$M_{m}\left(q,\;\xi_{b}\right)\ddot{q}+C_{m}\left(q,\;\dot{q},\;\xi_{b},\;{\dot{\xi }}_{b}\right)\dot{q}+g_{m}\left(q,\;\Phi_{b}\right)=\tau +J_{e}^{Τ}\left(q,\;\Phi_{b}\right)f_{ext}$$

where ***ξ****_b_* = [$p_{b}^{T}$ $\Phi_{b}^{T}$]^T^. ***τ*** = [*τ*_1_ *τ*_2_ *τ*_3_]^T^ is the joint torque vector of the robot arm, ***f****_ext_* is the interaction force exerted on the end effector from the environment. ***M****_m_* is the matrix of inertia terms, ***C****_m_* is of Coriolis and centrifugal terms, and ***g****_m_* is of gravity terms.

### 2.4. Environment

The aerial manipulator end effector will suffer contact force when it interacts with the environment. Considering our application purpose of side-on manipulation, a vertical wall along the side is supposed and modeled as a spring system with large stiffness [32], which will apply an orthogonal force to the end effector during contact. The environment model is described as

(14) $$f_{ext,y}:=\{\begin{array}{ll} -k_{wall}\left(y_{e}-y_{wall}\right) & \mathrm{if}\;y_{e}-y_{wall}>0\\ 0 & \mathrm{if}\;y_{e}-y_{wall}\leq 0 \end{array}$$

where *f_ext__,y_* is the force component in Y direction of the contact force vector ***f****_ext_*, *y_e_* is the position of the end effector in Y direction, *y_wall_* is the position of the vertical wall, and *k_wall_* is the stiffness of the wall.

## 3. Composite Controller Design of the Aerial Manipulator

### 3.1. Control System Overview

A typical application scenario of the aerial manipulator includes four stages [19]: takeoff and free flight, approaching and arm extension, contact and manipulation, retraction, and departure. There are two main challenges during this process. First, the aerial platform will be severely disturbed under the manipulator motion, which is far beyond ordinary disturbances such as wind gusts and the traditional controller cannot guarantee the robustness and stability of the system. Second, physical interaction of the end effector causes unexpected contact force, which may lead to damage or instability of the system, and thus the contact compliance must be ensured. To address these two challenges, a composite controller is proposed.

**Figure 3:**
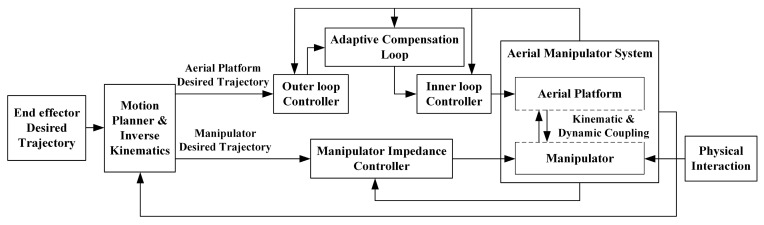
The configuration of the composite controller.

The overall structure of the control system is shown in Figure 3. For the given desired trajectory ***p****_e,d_* and the actual position ***p****_e_* of the aerial manipulator end effector, first, the desired references of the aerial vehicle position ***p****_b,d_* and yaw angle *ψ_d_* and the references of the end effector position relative to the vehicle body frame *^b^**p**_e,d_* are obtained through the motion planner module; then the aerial vehicle controller and manipulator controller are designed to track their desired trajectories and achieve stability of the system. A two-layer basic controller is used to achieve basic performance of the aerial platform, and an auxiliary adaptive controller is proposed to estimate and compensate the disturbances acting on the vehicle from the manipulator, which guarantees the stability of the vehicle under the manipulator motion. In addition, compared with the positioning accuracy of the floating aerial platform, the joint angles of the manipulator have relatively high control precision by off-the-shelf joint servos, and the response performance of the end-effector is highly dependent on the performance of the aerial platform. Therefore, the proposed auxiliary adaptive controller can also significantly improve the trajectory tracking performance of the overall aerial manipulator system. On the other hand, even with excellent vehicle position control, relative motions between the platform and the target highlight the need for compliant manipulation approaches, especially for the undesired collision situations. The proposed adaptive controller of the aerial platform has good anti-disturbance performance, but at the same time makes the system equivalent to a large stiffness system. Therefore, an impedance compliance controller of the manipulator is proposed to reduce the interaction force on the fuselage and maintain stability during the transition from free motion to interaction.

### 3.2. Aerial Vehicle Controller Design

**Figure 4:**
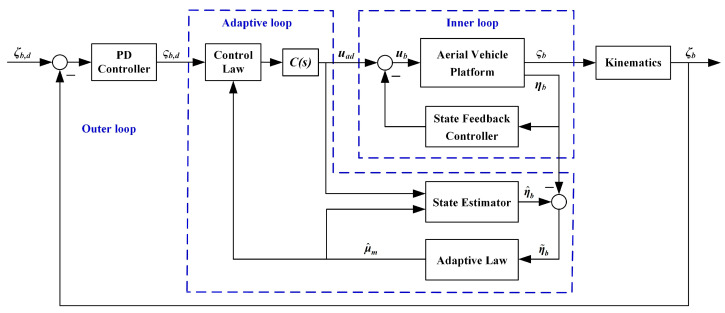
The configuration of the aerial platform controller.

**Figure 5:**
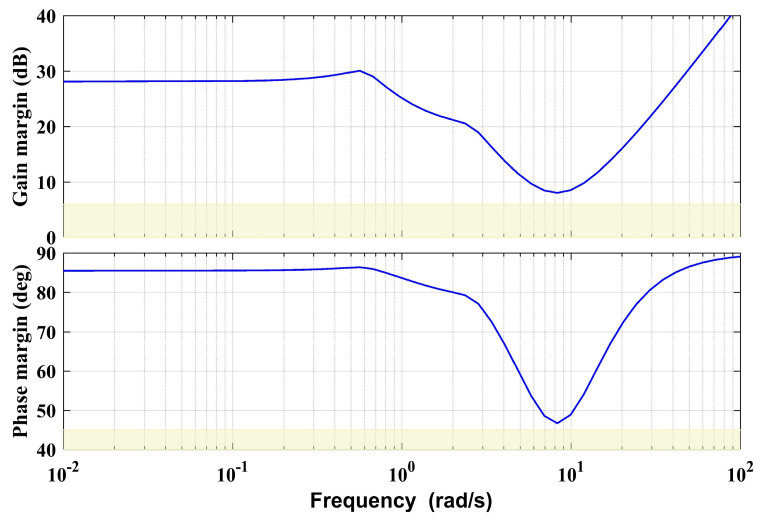
The stability margin of the inner loop system.

For the system described in Equation (4), a simple two-layer controller is applied for the basic control, as shown in Figure 4. Notice that for simplicity, let the vehicle position and yaw angle be denoted as ***ζ****_b_* = [*x_b_ y_b_ z_b_ ψ*]^T^, and ***ζ****_b,d_* and ***ς****_b,d_* are the desired references of ***ζ****_b_* and ***ς****_b_* accordingly. The inner loop is a state feedback-based controller, which is mainly used to realize the input–output decoupling and stabilization of the system. The outer loop is a series of PD controllers, which is for position tracking. The inner loop controller is designed as a robust H-infinity static controller, which is a widely used method and is not the focus of this paper. The main idea of the robust H-infinity control method is to transform the desired control performance indexes and robust stability conditions into H-infinity norm forms based on the H-infinity synthesis theory. Then the structural controller parameters can be tuned by solving the H-infinity norm optimization problem satisfying the stability margin constraint to ensure the stability and robust performance of the closed-loop system. The detailed theory and design process can be referred to in [33] and our previous work [31]. The stability margin of the closed loop system with the designed inner loop controller is shown in Figure 5. The stability margin is described by multivariable disk margin method, comprehensively considering all channels of the MIMO system. Figure 5 illustrates that the gain margin exceeds 6 dB, and the phase margin exceeds 45°, which shows that the inner loop controller can meet the basic performance and ensure the stability of the system. However, although the basic controller can achieve relatively good performance of the aerial vehicle, for the aerial manipulator integrated system, the extra robot arm leads to dramatic disturbances that would exceed the stability margin of the basic controller and the stability of the system cannot be guaranteed. Considering the existence of the manipulator, this section will focus on the auxiliary adaptive controller design to estimate and compensate for the impact of the manipulator.

The adaptive controller is designed based on the inner closed-loop system, as shown in Figure 4. The inner loop controller is a robust static controller, which does not increase the system order or change the physical meanings of the states, thus the inner closed-loop system can be described as

(15) $$\begin{array}{l} {\dot{\eta }}_{b}\left(t\right)=A_{bc}\eta_{b}\left(t\right)+B_{bc}u_{ad}\left(t\right)+\mu_{m}\left(t\right)\\ \varsigma_{b}\left(t\right)=C_{b}\eta_{b}\left(t\right) \end{array}$$

where ***A****_bc_* and ***B****_bc_* are the closed-loop state matrix and control matrix, ***u****_ad_* is the closed-loop system control input vector, and other variables are the same with Equation (4). Owing to the inner loop controller, the closed-loop system in Equation (15) is bounded-input bounded-state (BIBS) stable and ***A****_bc_* is Hurwitz. Moreover, all the state variables are measurable and ***η****_b_*(0) = **0**. ***μ**_m_*(*t*) is the time varying disturbance vector acting on the vehicle from the manipulator, which is bounded as ***μ****_m_*(*t*) ∈ 𝕄, namely, ∃M that ${\left\|\mu_{m}\left(t\right)\right\|}_{2}<M$ for all *t* ≥ 0, and further assume ***μ****_m_*(*t*) is differentiable with a bounded derivative. The boundary is estimated by the arm dynamics acting on the vehicle and multiplied by a relaxation factor of 1.2, supposing that the arm extends to its extreme position while the joint has maximum acceleration. Notice that such a case is almost impossible in practice, namely, the disturbance boundary is much larger than the actual disturbance in the real manipulation process. However, the loose boundary assumption can ensure the effectiveness of the adaptive controller.

The adaptive controller includes three parts. First, a state estimator is designed for state estimation; then according to the estimation error, an adaptive law is designed to estimate the disturbance ***μ****_m_*; finally, based on the disturbance estimation and the output of the outer loop PD controller ***ς****_b,d_*, the control law ***u****_ad_* is calculated as the new reference input of the inner loop system to compensate the disturbance.

For the system described in Equation (15), the state predictor is designed as

(16) $$\begin{array}{l} {\dot{\hat{\eta }}}_{b}\left(t\right)=A_{bc}{\hat{\eta }}_{b}\left(t\right)+B_{bc}u_{ad}\left(t\right)+{\hat{\mu }}_{m}\left(t\right)\\ {\hat{\varsigma }}_{b}\left(t\right)=C_{b}{\hat{\eta }}_{b}\left(t\right) \end{array}$$

where the variables with cap ^ refer to their estimation accordingly, and the estimation error of ***η****_b_* and ***μ****_m_* can be calculated as

(17) $$\begin{array}{l} {\tilde{\eta }}_{b}\left(t\right)={\hat{\eta }}_{b}\left(t\right)-\eta_{b}\left(t\right)\\ {\tilde{\mu }}_{m}\left(t\right)={\hat{\mu }}_{m}\left(t\right)-\mu_{m}\left(t\right) \end{array}$$

Based on the state estimation, let the adaptive law of disturbance estimation be

(18) $${\dot{\hat{\mu }}}_{m}\left(t\right)=\Gamma \mathrm{Proj}\left({\hat{\mu }}_{m}\left(t\right),\;-{\left({\tilde{\eta }}_{b}^{T}\left(t\right)P_{bc}\right)}^{T}\right)$$

where ${\tilde{\eta }}_{b}\left(t\right)$ is the estimation error defined in Equation (17), Γ is the adaptive gain. **Proj**(·, ·) is the projection operator, which is a piecewise function and used to guarantee the boundedness of the disturbance estimation when the estimation is tend to divergent and keep the estimation within the given boundary. Let $y_{proj}(t):=-{\left({\tilde{\eta }}_{b}^{T}\left(t\right)P_{bc}\right)}^{T}$, the projection operator is defined as

(19) $$\begin{array}{l} \mathrm{Proj}\left({\hat{\mu }}_{m}\left(t\right),\;y_{proj}(t)\right):=\\ \{\begin{array}{ll} y_{proj}(t) & {\hat{\mu }}_{m}\left(t\right)\in S1\\ y_{proj}(t) & {\hat{\mu }}_{m}\left(t\right)\in S2\\ y_{proj}(t)-\frac{\nabla f\left({\hat{\mu }}_{m}\left(t\right)\right)}{\left\|\nabla f\left({\hat{\mu }}_{m}\left(t\right)\right)\right\|}\langle \frac{\nabla f\left({\hat{\mu }}_{m}\left(t\right)\right)}{\left\|\nabla f\left({\hat{\mu }}_{m}\left(t\right)\right)\right\|},\;y_{proj}(t)\rangle f\left({\hat{\mu }}_{m}\left(t\right)\right) & {\hat{\mu }}_{m}\left(t\right)\in S3 \end{array} \end{array}$$

where $f\left({\hat{\mu }}_{m}\left(t\right)\right):=\left(\left(\varepsilon_{\mu }+1\right){\hat{\mu }}_{m}^{T}\left(t\right){\hat{\mu }}_{m}\left(t\right)-M^{2}\right)\cdot {\left(\varepsilon_{\mu }M^{2}\right)}^{-1}$, *ε_μ_* > 0 is the projection tolerance bound, $\nabla f\left({\hat{\mu }}_{m}\left(t\right)\right)$ is the gradient vector of f evaluated at ${\hat{\mu }}_{m}$. S1, S2, and S3 are defined as

(20) $$\begin{array}{l} S1:=\left\{{\hat{\mu }}_{m}\left(t\right)|f\left({\hat{\mu }}_{m}\left(t\right)\right)<0\right\}\\ S2:=\left\{{\hat{\mu }}_{m}\left(t\right)|f\left({\hat{\mu }}_{m}\left(t\right)\right)\geq 0\wedge \nabla f^{T}\left({\hat{\mu }}_{m}\left(t\right)\right)y_{proj}(t)\leq 0\right\}\\ S3:=\left\{{\hat{\mu }}_{m}\left(t\right)|f\left({\hat{\mu }}_{m}\left(t\right)\right)\geq 0\wedge \nabla f^{T}\left({\hat{\mu }}_{m}\left(t\right)\right)y_{proj}(t)>0\right\} \end{array}$$

The detailed explanation of the projection operator can be referred to in [34]. ***P****_bc_* is the solution of the algebraic Lyapunov equation of the system in Equation (15), which is given as

(21) $$A_{bc}^{T}P_{bc}+P_{bc}A_{bc}=-I$$

where ***I*** is the identity matrix. Then based on the reference input ***ς****_b,d_* and the estimation of the disturbance ***μ****_m_*, the new control signal can be reconstructed to track the reference ***ς****_b,d_* whilst compensate the disturbance ***μ****_m_*, which is as follow with Laplace form:

(22) $$u_{ad}\left(s\right)=C\left(s\right)\left(K_{des}\varsigma_{b,d}\left(s\right)-F\left(s\right){\hat{\mu }}_{m}\left(s\right)\right)$$

where ***K****_des_* = −(***C****_b_*$A_{bc}^{-1}$***B****_bc_*)^−1^ is to ensure that ***ς****_b_* can track ***ς****_b,d_* with zero steady-state error, and ***F***(*s*) = $H_{b}^{-1}$(*s*)***C****_b_**H**_bη_*(*s*), where ***H****_bη_*(*s*) = (*s**I*** − ***A****_bc_*)^−1^, ***H****_buη_*(*s*) = ***H****_bη_*(*s*)***B****_bc_*, ***H****_b_*(*s*) = ***C****_b_**H**_buη_*(*s*). Notice that a low-pass filter matrix ***C***(*s*) is applied to shape the control input before giving to the inner loop of the system. The use of ***C***(*s*) is inspired by the theory in [35], which decouples the adaptive rate from the robustness, and therefore the fast adaption estimation speed can be applied by increasing the adaptive gain to guarantee the disturbance estimation accuracy, whilst avoiding an adverse effect on the states and ensuring the robustness. According to [35], the choice of ***C***(*s*) should satisfy the following conditions:

(23) $$\begin{array}{l} C(s)\;\mathrm{is}\;\mathrm{strictly}\;\mathrm{proper}\;\mathrm{and}\;\mathrm{stable}\;\mathrm{with}\;C(0)=I\\ \exists \varepsilon_{1},\;{\left\|C(s)H_{b}^{-1}(s)\right\|}_{L_{1}}<\varepsilon_{1}\\ \exists \varepsilon_{2},\;{\left\|H_{b\eta }(s)\left(I-B_{bc}C(s)F(s)\right)\right\|}_{L_{1}}<\varepsilon_{2} \end{array}$$

where the subscript *L*_1_ denotes the *L*_1_ norm, and the last two conditions indicate that the corresponding *L*_1_ norm gains are finite.

In order to perform the stability and performance analyses of the proposed adaptive controller, first, consider the stability of the state estimator. The error dynamics of the state estimator can be described through Equations (15)–(17) as

(24) $${\dot{\tilde{\eta }}}_{b}\left(t\right)=A_{bc}{\tilde{\eta }}_{b}\left(t\right)+{\tilde{\mu }}_{m}\left(t\right)$$

Considering the following Lyapunov candidate function:

(25) $$V_{b}\left({\tilde{\eta }}_{b}\left(t\right),\;{\tilde{\mu }}_{m}\left(t\right)\right)={\tilde{\eta }}_{b}^{T}\left(t\right)P_{bc}{\tilde{\eta }}_{b}\left(t\right)+\Gamma^{-1}{\tilde{\mu }}_{m}^{T}\left(t\right){\tilde{\mu }}_{m}\left(t\right)$$

and considering the disturbance boundary one has

(26) $$V_{b}\left(0\right)=\Gamma^{-1}{\tilde{\mu }}_{m}^{T}\left(0\right){\tilde{\mu }}_{m}\left(0\right)\leq \Gamma^{-1}\underset{t\geq 0}{\max}\left({\tilde{\mu }}_{m}^{T}\left(t\right){\tilde{\mu }}_{m}\left(t\right)\right)<4\Gamma^{-1}M^{2}$$

where M is the norm boundary of the disturbance ***μ****_m_*. Taking the derivation of Equation (25) and combined with Equations (18), (21), and (24) one has

(27) $$\begin{array}{ll} {\dot{V}}_{b}\left(t\right) & =-{\tilde{\eta }}_{b}^{T}\left(t\right){\tilde{\eta }}_{b}\left(t\right)+2{\tilde{\mu }}_{m}^{T}\left(t\right)\left({\left({\tilde{\eta }}_{b}^{T}\left(t\right)P_{bc}\right)}^{T}+\mathrm{Proj}\left({\hat{\mu }}_{m}\left(t\right),\;-{\left({\tilde{\eta }}_{b}^{T}\left(t\right)P_{bc}\right)}^{T}\right)\right)\\ & =-{\tilde{\eta }}_{b}^{T}\left(t\right){\tilde{\eta }}_{b}\left(t\right)\leq 0 \end{array}$$

and it follows that for all *t* ≥ 0,

(28) $$V_{b}\left(t\right)\leq V_{b}\left(0\right)<4\Gamma^{-1}M^{2}$$

Thus the state estimation error dynamics is Lyapunov stable and the estimation error of ***η****_b_* is uniformly bounded, given as

(29) $${\left\|{\tilde{\eta }}_{b}(t)\right\|}_{2}\leq \sqrt{\lambda_{\min}^{-1}(P_{bc})\left({\tilde{\eta }}_{b}^{T}\left(t\right)P_{bc}{\tilde{\eta }}_{b}\left(t\right)\right)}\leq \sqrt{4\lambda_{\min}^{-1}(P_{bc})\Gamma^{-1}M^{2}}$$

where *λ*_min_(***P****_bc_*) is the minimum eigenvalue of ***P****_bc_*. It can also be drawn from Equation (29) that the boundary of state estimation error is inversely proportional to the square root of the adaptive gain Γ, which denotes that the performance of the state estimator can be improved by increasing the adaptive gain.

Next, consider the following ideal form of the adaptive closed-loop system, in which the designed controller can estimate and compensate the disturbance perfectly:

(30) $$\begin{array}{l} {\dot{\eta }}_{br}\left(t\right)=A_{bc}\eta_{br}\left(t\right)+B_{bc}u_{r}\left(t\right)+\mu_{m}\left(t\right)\\ \varsigma_{br}\left(t\right)=C_{b}\eta_{br}\left(t\right) \end{array}$$

where the control input has an ideal form as ***u****_r_*(*s*) = ***C***(*s*)(***K****_des_**ς**_b,d_*(*s*) − ***F***(*s*)***μ****_m_*(*s*)) and ***η****_br_*(0) = **0**. Describe Equation (30) in Laplace form as

(31) $$\eta_{br}\left(s\right)=H_{bu\eta }(s)C\left(s\right)K_{des}\varsigma_{b,d}\left(s\right)+H_{b\eta }(s)\left(I-B_{bc}C\left(s\right)F\left(s\right)\right)\mu_{m}\left(s\right)$$

Since ***A****_bc_* is Hurwitz, ***μ****_m_* is bounded, and the conditions in Equation (23) are held, one has that the *L*_1_ norm of the ideal system in Equation (31) is bounded for all *t* ≥ 0. Using the *L*_1_ norm stability theorem [35], the ideal system of Equation (30) is BIBS stable and ${\left\|\eta_{br}\right\|}_{L_{\infty }}$s uniformly bounded. The performance bound of the system can be given as

(32) $${\left\|\eta_{br}\right\|}_{L_{\infty }}\leq {\left\|H_{bu\eta }(s)C\left(s\right)K_{des}\right\|}_{L_{1}}{\left\|\varsigma_{b,d}\right\|}_{L_{\infty }}+{\left\|H_{b\eta }(s)\left(I-B_{bc}C\left(s\right)F\left(s\right)\right)\right\|}_{L_{1}}{\left\|\mu_{m}\right\|}_{L_{\infty }}$$

where the subscript *L*_1_ denotes the *L*_1_ norm and the subscript *L*_∞_ denotes the *L*_∞_ norm.

Similar to the ideal closed-loop system, describe the real closed-loop system Equation (15) in Laplace form as

(33) $$\begin{array}{l} \eta_{b}\left(s\right)=H_{bu\eta }(s)C\left(s\right)K_{des}\varsigma_{b,d}\left(s\right)+H_{b\eta }(s)\left(I-B_{bc}C\left(s\right)F\left(s\right)\right)\mu_{m}\left(s\right)\\ \;\;\;-H_{bu\eta }(s)C\left(s\right)F\left(s\right){\tilde{\mu }}_{m}\left(s\right) \end{array}$$

Combined with Equations (24), (31), and (33) and considering the boundary of state estimation error in Equation (29) the adaptive state error between the ideal adaptive system and the real adaptive system can be proved uniformly bounded by *L*_1_ norm stability theorem [35] as

(34) $$\begin{array}{ll} {\left\|e_{\eta b}\right\|}_{L_{\infty }} & ={\left\|\eta_{br}-\eta_{b}\right\|}_{L_{\infty }}\leq {\left\|H_{bu\eta }(s)C\left(s\right)H_{b}^{-1}(s)C_{b}\right\|}_{L_{1}}{\left\|{\tilde{\eta }}_{b}\right\|}_{L_{\infty }}\\ & \leq {\left\|H_{bu\eta }(s)C\left(s\right)H_{b}^{-1}(s)C_{b}\right\|}_{L_{1}}\sqrt{4\lambda_{\min}^{-1}(P_{bc})\Gamma^{-1}M^{2}} \end{array}$$

Similarly, the adaptive input error can be proved uniformly bounded:

(35) $${\left\|e_{ub}\right\|}_{L_{\infty }}={\left\|u_{r}-u_{ad}\right\|}_{L_{\infty }}\leq {\left\|C\left(s\right)H_{b}^{-1}(s)C_{b}\right\|}_{L_{1}}\sqrt{4\lambda_{\min}^{-1}(P_{bc})\Gamma^{-1}M^{2}}$$

Considering Equations (32), (34), and (35) synthetically, it can be derived that when there is disturbance acting on the vehicle from the manipulator, the closed-loop system of the vehicle with the proposed adaptive controller is BIBS stable. Additionally, since the use of ***C***(*s*) decouples the adaptive rate from the robustness of the system, the performance of the controller can be improved by large adaptive gain.

Stated thus, for the system of Equation (15), the adaptive controller is designed via the state estimator of Equation (16), adaptive law of Equation (18), and control law of Equation (22) by choosing appropriate adaptive gain Γ and filter matrix ***C***(*s*) with satisfying conditions of Equation (23). According to the controller performance analysis above, a large adaptive gain Γ can be adopted to improve the tracking performance of the controller, which is only limited by the computing power of the hardware. The use of the bandwidth-limited filter matrix ***C***(*s*) is to realize the reference tracking and disturbance compensation within the specified frequency range. The increase of the bandwidth of ***C***(*s*) can improve the tracking performance to an ideal system, however, it may lead to the reduced time-delay margin and hurt the robustness of the closed-loop system. Following the method in [35], on the basis of satisfying conditions of Equation (23), the parameters of ***C***(*s*) can be obtained after a series of adjustments and iterations to achieve good control performance. With above analysis, the control parameters of the proposed controller are designed as

(36) $$\begin{array}{l} C(s)=\frac{25}{s^{2}+6s+25}I\\ \Gamma =1000 \end{array}$$

Moreover, the solution ***P****_bc_* of the algebraic Lyapunov equation in Equation (21) is given as

(37) $$P_{bc}=\left(\begin{array}{ccccccccc} 0.6062 & 0.0007 & 0.0008 & 0.0003 & -0.0647 & 0.0005 & -0.0050 & -1.0889 & 0.0008\\ 0.0007 & 0.6056 & 0.0004 & 0.0613 & -0.0002 & -0.0071 & 1.0841 & -0.0009 & -0.0006\\ 0.0008 & 0.0004 & 0.4996 & 0.0012 & -0.0034 & -0.0018 & -0.0012 & -0.0007 & -0.0012\\ 0.0003 & 0.0613 & 0.0012 & 0.0667 & -0.0010 & -0.0077 & 0.1444 & 0.0003 & -0.0076\\ -0.0647 & -0.0002 & -0.0034 & -0.0010 & 0.0711 & -0.0010 & 0.0011 & 0.1505 & -0.0013\\ 0.0005 & -0.0071 & -0.0018 & -0.0077 & -0.0010 & 0.0639 & 0.0489 & \;0.0101 & 0.0572\\ -0.0050 & 1.0841 & -0.0012 & 0.1444 & 0.0011 & 0.0489 & 6.0635 & -0.0301 & 0.0358\\ -1.0889 & -0.0009 & -0.0007 & 0.0003 & 0.1505 & 0.0101 & -0.0301 & 6.0291 & 0.0074\\ 0.0008 & -0.0006 & -0.0012 & -0.0076 & -0.0013 & 0.0572 & 0.0358 & 0.0074 & 1.0575 \end{array}\right)$$

### 3.3. Manipulator Controller Design

To perform operation tasks, the aerial manipulator system not only needs to guarantee good positioning and tracking performance as the traditional UAV does, but also needs to be in contact with the environment. However, physical contact poses great challenges to the stability of the system, especially when the undesired collision occurs. Different from the ordinary base-fixed industrial manipulator system, the relative motion between the floating platform and the target of the aerial manipulator system is inevitable, and the reaction force that the floating platform can bear is limited, which illustrates the importance of the compliance during the contact process. Thus, an impedance strategy of the manipulator is designed, pulling the end effector to the desired position as a virtual spring-damper system. The end effector behaves in a compliant manner during contact to reduce the reaction force and absorb the impact on the fuselage.

The architecture of the impedance controller is illustrated in Figure 3. For classic industrial manipulators, impedance control based on the force measurement and feedback is widely used [36]. However, for the aerial manipulator, it is difficult to accurately measure the contact force of the end effector due to the drift of the aerial platform and the unpredictable disturbances such as unmodeled aerodynamic effects. Considering that the main purpose of the controller is to achieve contact compliance, the position-based impedance control method proposed in [37] was adopted. For the manipulator system described in Equation (13), define the impedance control law as

(38) τ=JeΤ(q, Φb)(Mep¨e,db+Cep˙e,db+KD(p˙e,db−p˙eb)+KP(pe,db−peb))+gm(q, Φb)

where ***τ*** = [*τ*_1_ *τ*_2_ *τ*_3_]^T^ is the joint torque vector of the arm. *^b^**p**_e,d_* is the desired reference of the position of the end effector with respect to the vehicle, which is obtained from the motion planner module, and *^b^**p**_e_* is the actual response accordingly. ***K****_D_* and ***K****_P_* are the desired damping and stiffness matrices to achieve desired controller behavior. ***M****_e_* and ***C****_e_* are the matrices of inertia terms and Coriolis terms with respect to *^b^**p**_e_*, which are functions of the inertia matrix, Coriolis matrix, and Jacobian matrix of arm joints, obtained as

(39) $$\begin{array}{l} M_{e}=J_{e}^{-T}\left(q,\;\Phi_{b}\right)M_{m}\left(q,\;\xi_{b}\right)J_{eb}^{-1}\left(q\right)\\ C_{e}=J_{e}^{-T}\left(q,\;\Phi_{b}\right)\left(C_{m}\left(q,\;\dot{q},\;\xi_{b},\;{\dot{\xi }}_{b}\right)-M_{m}\left(q,\;\xi_{b}\right)J_{eb}^{-1}\left(q\right){\dot{J}}_{eb}\left(q\right)\right)J_{eb}^{-1}\left(q\right) \end{array}$$

Combined with Equations (7), (13) and (38), the closed-loop system behavior with the impedance controller can be described as

(40) Mep˜¨eb+(Ce+KD)p˜˙eb+KPp˜eb=fextp˜eb=pe,db−peb

The stability of the system in Equation (40) can be proved by considering the following Lyapunov candidate function:

(41) Vm(p˜˙eb, p˜eb)=12p˜˙eTbMep˜˙eb+12p˜eTbKPp˜eb

and the detailed proof process can be referred to in [37] and [38]. ***K****_D_* and ***K****_P_* can be tuned following the method in [37], given as ***K****_D_* = 85***I*** and ***K****_P_* = 100***I***.

### 3.4. Simulation Analyses

To validate the effectiveness of the proposed controllers in Section 3.2 and Section 3.3, the simulations were carried out. The tracking tests of the aerial platform under the manipulator motion were simulated to verify the adaptive controller of the aerial platform, and the contact tests were simulated to verify the impedance controller of the manipulator. In the simulations, the nonlinear model with the real system parameters proposed in Section 2 was used, and the controller parameters can refer to the corresponding controller design sections.

#### 3.4.1. Flight Test Simulation

**Figure 6:**
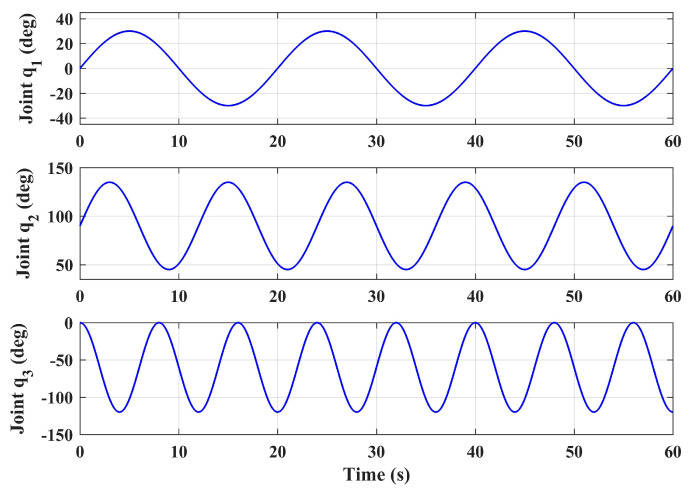
Joint angles in flight test simulation.

The step tracking responses of the position and yaw angle of the aerial platform were simulated under the large swing references of the manipulator joint angles. The initial angles of the three joints of the manipulator were ***q***_0_ = [0° 90° 0°]^T^, and in this condition the center of gravity of the manipulator was located on the Z*_b_* axis, which is a symmetrical position relative to the vehicle body-fixed coordinate frame. Let the three joint angles make sinusoidal motions around the initial positions with the swing ranges of [−30°, 30°], [45°, 135°] and [−120°, 0°] respectively, and give different reference frequencies to ensure the variability of the forces act on the body. The three joint angles are given in Equation (42), and illustrated in Figure 6. Notice that the motion range and frequency given here are severe, which exceeds the motion requirements in general operation tasks, and can characterize the worst case. The joint 1 has a relatively small swing angle, which is due to the limitation of the prototype structure on the motion range of joint 1. Since the aim of the proposed design is to achieve the side-on manipulation, the motion range of joint 1 is reasonable.

(42) $$\begin{array}{l} q_{1,d}=\frac{\pi }{6}\sin\left(\frac{\pi }{10}t\right)\\ q_{2,d}=\frac{\pi }{4}\sin\left(\frac{\pi }{6}t\right)+\frac{\pi }{2}\\ q_{3,d}=\frac{\pi }{3}\sin\left(\frac{\pi }{4}t+\frac{\pi }{2}\right)-\frac{\pi }{3} \end{array}$$

**Figure 7:**
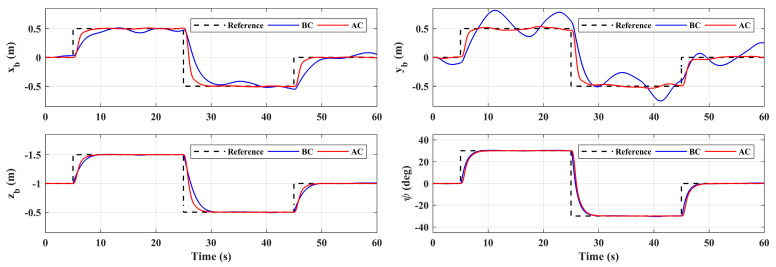
System responses under manipulator motion.

**Figure 8:**
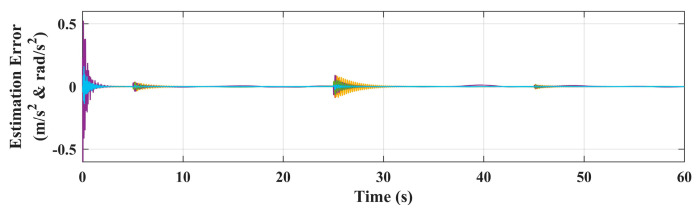
Estimation error of the adaptive controller.

The simulation results of the responses of *x_b_*, *y_b_*, *z_b_,* and *ψ* are shown in Figure 7. As a comparison, the system responses with the basic controller (without adaptive loop) are also given in Figure 7, where BC denotes the basic controller and AC denotes the controller with adaptive auxiliary loop. The proposed adaptive controller can guarantee the tracking performance of all four channels. As a comparison, due to the manipulator motion, the responses deviate from the reference dramatically without the adaptive loop. Among them, the *y_b_* channel is the most affected, and the maximum deviation reaches 70% from the reference, which is obviously unable to meet the task requirements. The fluctuation of the *x_b_* channel is the second, and the influence on the *z_b_* and *ψ* channel are relatively small. This is because the arm extends from the side of the body, thus its movement mainly affects the lateral channel. Moreover, when the angle of joint 1 is not 0, it will also have an impact on the longitudinal channel. The disturbance estimation error of the proposed adaptive controller is also given in Figure 8, which shows that the controller has good estimation accuracy.

#### 3.4.2. Contact Test Simulation

**Figure 9:**
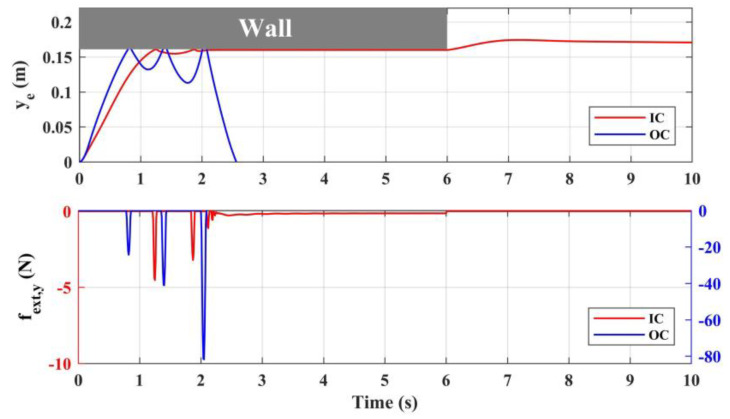
System responses during contact.

Given the position reference of the manipulator end effector ***p****_e,d_* to extend the arm from the side-on and contact the vertical wall, the responses of end effector position *y_e_* and contact force *f_ext__,y_* during the contact are illustrated in Figure 9. In this process, the aerial platform remains to hover, the end effector references in longitudinal and altitudinal directions remain stationary, and let the end effector extend along the lateral direction. The wall is assumed to disappear in the 6th second. The wall model used in simulation is described in Section 2.4, where the stiffness of the wall is set as *k_wall_* = 10,000. As a comparison, the system responses with the ordinary PID controller are also given in Figure 9, where IC denotes the impedance controller, and OC denotes the ordinary controller. In the force response subfigure, the ordinate axis corresponding to IC is set on the left side with red, while which corresponding to OC is set on the right with blue.

The results show that without the impedance controller, the great impact force was produced when the end effector contacts the wall, where the initial impact force was higher than 20 N, and the system gradually diverged and became unstable after three impacts. This is because the aerial platform of the aerial manipulator is floating, thus compared with the fixed-base industrial manipulator, the reaction force from the end effector will have a great impact on the base platform. Moreover, the acceleration and velocity of the aerial platform will further increase the impact force. In fact, in practice the initial contact force has already exceeded the maximum endurance of the manipulator, which would lead to the damage of the joint actuator, indicating the necessity of compliance control of the manipulator. The proposed impedance controller can realize the stable compliance contact between the end effector and the wall, where the initial impact force is less than 5 N, and then quickly attenuates after several docking processes. In order to fully consider the sudden change of environment, after the physical collision, the wall is also assumed to disappear in the 6th second. From the results, the contact force became 0, and the end effector could reach a balance at the reference position behind the wall, indicating that the system could ensure safety in both situations, which verifies the effectiveness of the design of the manipulator impedance controller.

## 4. Experimental Tests and Verification

### 4.1. Experiment Description

**Figure 10:**
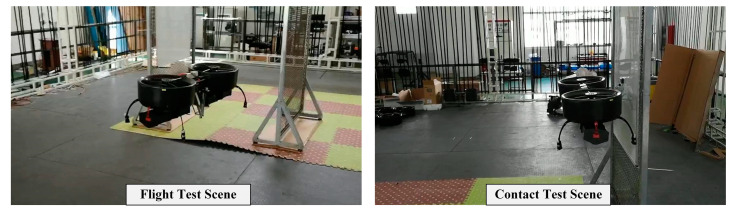
The scenes of the experimental tests.

The experimental tests were carried out to evaluate the performance of the proposed controller. Considering the two main challenges during the aerial manipulation process mentioned in Section 1, two kinds of experiments were performed accordingly. First, considering the arm extension stage, the hovering and tracking flight tests under manipulator motion were carried out to verify the stability and positioning performance; second, considering the physical interaction stage, the contact test of the end effector with a vertical wall was carried out to verify the stability and compliance performance of the system during continuous physical interaction. The two scenes are illustrated in Figure 10.

**Figure 11:**
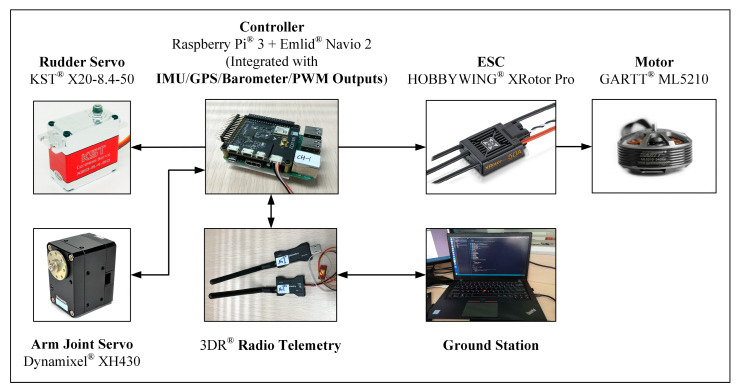
The illustration of the electronic hardware system.

Figure 11 illustrates the electronic hardware system. The on-board controller was Emlid^®^ Navio 2 (Emlid Co., HongKong), which was combined with Raspberry Pi^®^ 3 (Raspberry Pi Foundation, Cambridge, UK), integrating the dual IMU module, barometer module, GPS module, and PWM output channels. The motors of ducted fans were GARTT^®^ ML5210 (GARTT Co., Shenzhen, China), driven by HOBBYWING^®^ Electronic Speed Controller (ESC) (HOBBYWING Co., Shenzhen, China). The two rudder systems were driven by two KST^®^ X20 servos (KST Digital Technology Co., Shenzhen, China) respectively. Notice that the rudder system below one duct was separated by the fairing in the middle, but the two parts of it were driven synchronously by the same servo, which were considered as one set. The manipulator joint servo is Dynamixel^®^ XH430 (ROBOTIS Co., Seoul, Korea), which had both the position control mode and torque control mode, and could feedback both the position, velocity, and torque of the joint. Moreover, the OptiTrack^®^ (NaturalPoint Co., Corvallis, OR, USA) indoor positioning system was used to facilitate indoor tests.

### 4.2. Flight Test Results and Analyses

In the flight test, two scenarios are carried out. Scenario 1 involves the hovering test under the random manipulator motion, which refers to the comprehensive evaluation of the system under the general situation. Scenario 2 involves the tracking test under the large sinusoidal manipulator motion, which refers to the extreme cases of the system.

**Figure 12:**
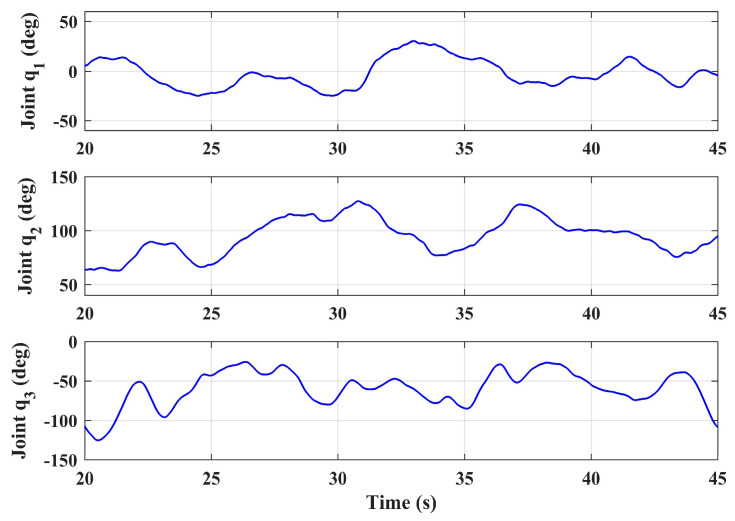
Joint angles in the hovering test.

In scenario 1, let the aerial manipulator hover at the height of 1 m (the references of *x_b_*, *y_b_,* and *ψ* are 0 and the reference of *z_b_* is −1), and let the manipulator joint motion randomly, obtaining the position and attitude responses of the aerial platform. As a comparison, the responses of the basic controller with the same manipulator motion were also given. The responses of the joint angles of the arm during the experiment are shown in Figure 12. Notice that the main concern here was the system stability performance under the movement of the manipulator, not the end effector position of the manipulator. Therefore, the reference input of the manipulator was direct to the joint angle rather than the end effector position, so as to ensure each joint of the manipulator had a large motion range.

**Figure 13:**
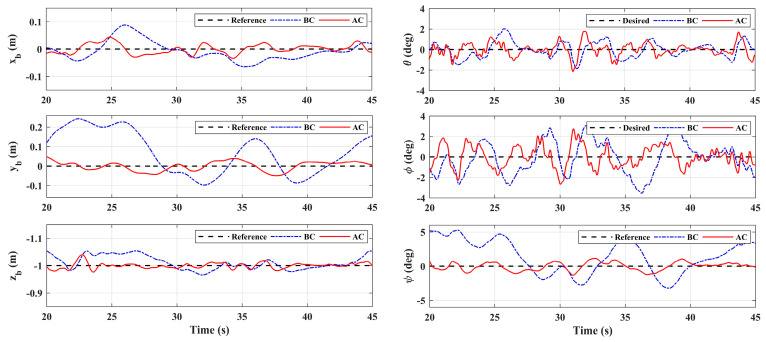
System responses in Scenario 1.

It can be seen from Figure 13 that the motion of the manipulator had a significant impact on the hovering accuracy of the aerial platform, the performance of the basic controller could not meet the task requirements, while the proposed adaptive controller could effectively improve the system’s performance. The influence of the arm on the lateral position *y_b_* of the aerial platform was the greatest, and the maximum deviation along the lateral direction was approximately +0.25 m. This is because the arm extended out from the body in this direction, and the link 2 and link 3 of the arm generated large gravity moment and inertia moment. As a comparison, the fluctuation error was reduced to ±0.05 m with adaptive loop compensation. When joint 1 of the manipulator was not in its initial position, the movement of the manipulator also had an influence on *x_b_*, the fluctuation range of the longitudinal position was 0.14 m, but it was less than 0.08 m with the adaptive controller. The system had the highest control performance in the altitude channel, the hovering errors were within ±0.05 m and ±0.04 m respectively without and with the adaptive loop. The inertial force produced by joint 1 also had an impact on yaw channel, and the maximum deviation of yaw angle *ψ* decreased from +5 to +2° with the adaptive compensation. Table 3 shows the root mean square error (RMSE) values of the position and attitude angle of the aerial platform. With the proposed adaptive controller, the performance of *y_b_* channel was improved by 81%, and the performance of *x_b_* and *z_b_* were also improved by around 50%, which illustrates the effectiveness of the designed controller.

**Figure 14:**
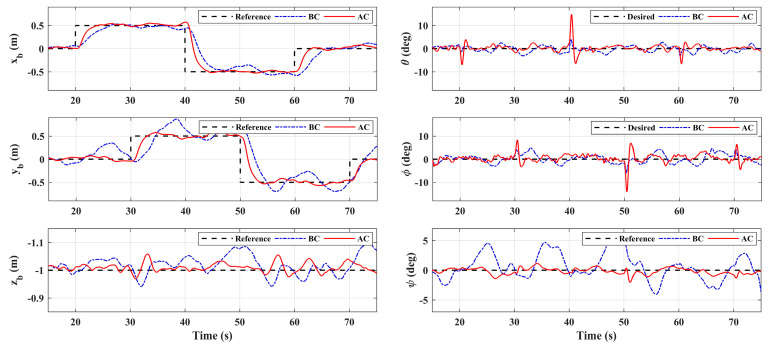
System responses in Scenario 2.

**Figure 15:**
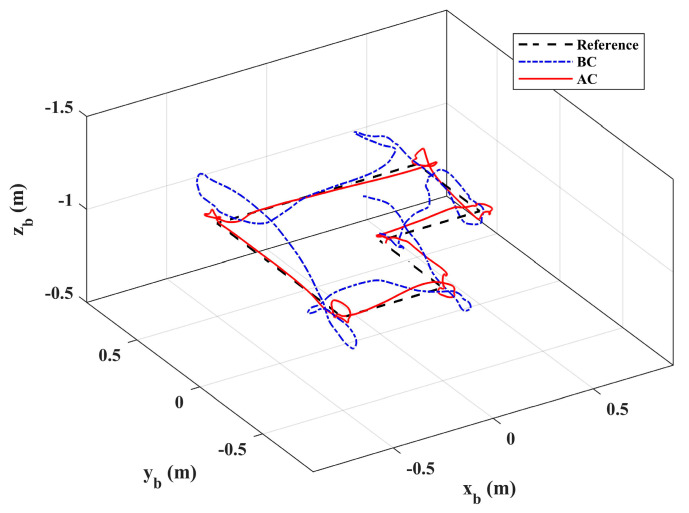
3D trajectory in Scenario 2.

**Figure 16:**
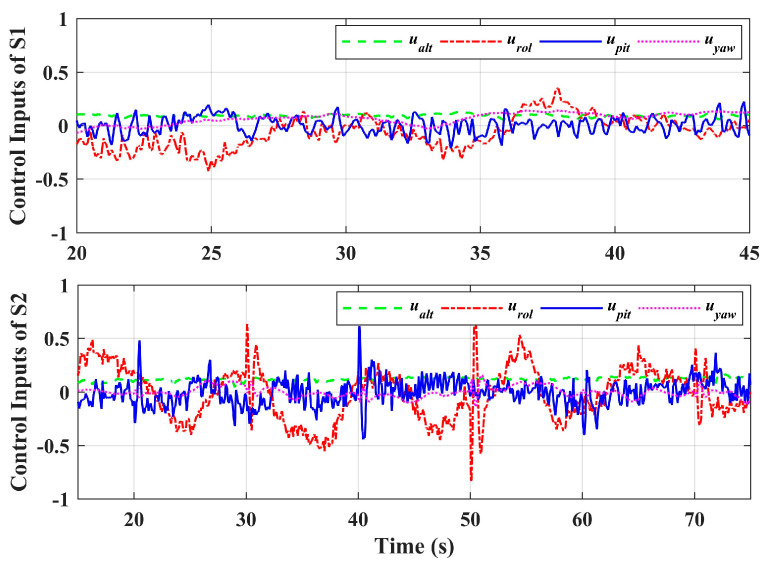
Control inputs of Scenario 1 and Scenario 2.

In scenario 2, let *x_b_* and *y_b_* do the point tracking test to verify the tracking performance of the system. During the test, keep the platform at the fixed height (same as scenario 1) for safety. Additionally, let the manipulator perform a large sinusoidal motion, as shown in Figure 6, which exceeds the motion requirements in general operation tasks, to evaluate system performance in extreme cases. A comparison with the basic controller was also carried out. Figure 14 illustrates the system responses and Figure 15 gives the 3D trajectory during tracking. The results show that the system with adaptive controller had good tracking performance, and the tracking accuracy could be guaranteed even when the manipulator moved in a large range. However, the response errors of the system were larger than that of scenario 1, which was due to the more violent motion of the manipulator, and the maneuvering of the aerial platform. On the other hand, the system responses with the basic controller had large errors, which could not meet the basic requirement of the system. Table 4 shows the RMSE values in the tracking test. Notice that the RMSEs here could not be compared directly with Table 3, because the desired references that were being tracked were changing. However, the comparison between the basic controller and the adaptive controller shows the effectiveness of the designed controller. Notice that the RMSEs of roll angle and pitch angle were larger, which is due to the constant adjustment of the attitude angles for the fast tracking response and real-time disturbance compensation. Moreover, Figure 16 shows the system control inputs of scenario 1 and scenario 2 respectively, which also illustrates that scenario 2 had greater challenges.

### 4.3. Contact Test Results and Analyses

**Figure 17:**
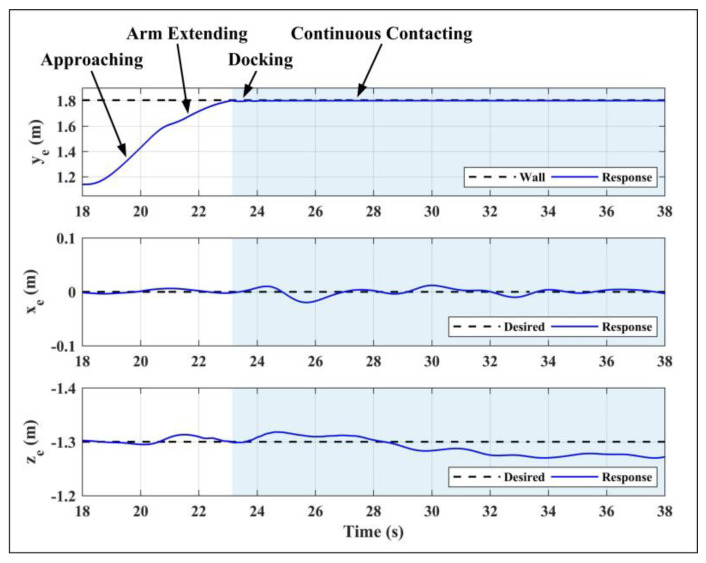
End effector position responses in Scenario 3.

**Figure 18:**
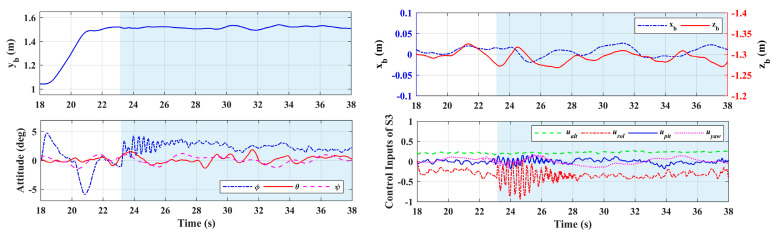
Aerial platform responses in Scenario 3.

The contact test was carried out in scenario 3. Set the vertical wall at the lateral direction of +1.8 m, and give the desired position references of the end effector to drive the end effector approach and contact the wall along the lateral direction, while *x_e,d_* and *z_e,d_* remained stationary. In order to achieve stable contact between the end effector and the wall, the lateral reference position *y_e,d_* was set at 1 cm behind the wall. During the test, for the given references of the end effector, the references of the aerial platform and the manipulator were obtained by the motion planner module in the controller. First, the aerial platform was driven to fly near the wall so that the distance between the end effector and the wall was less than 0.2 m, then the platform remained hovering and the arm extended forward to achieve physical contact. Figure 17 shows the end effector’s position responses. The whole process can be divided into four stages: aerial platform approaching, manipulator extending, docking, and continuous contacting. The right half blue part in Figure 17 and also Figure 18 denotes the interaction process with the wall. Notice that for safety, the test based on the ordinary PID controller was not carried out, only the test based on the impedance controller was performed.

As shown in Figure 17, the proposed impedance controller could realize the stable compliance contact, and the fluctuation errors of *x_e_* and *z_e_* during the contact were less than ±0.02 m and ±0.03 m respectively. Notice that the position control accuracy of the end effector here was higher than that of the aerial platform in scenario 1 and scenario 2, which was because the motion of the manipulator during the interaction process was simple with a small range and single direction, while that was violent in scenario 1 and scenario 2. On the other hand, the manipulator controller could compensate for the deviation caused by the floating platform to ensure the high-precision performance of the end effector. Figure 18 shows the responses of the aerial platform. The subfigure 1 refers to the lateral position of the platform, and the subfigure 2 refers to the longitudinal and altitude positions, where the ordinate axis corresponding to *x_b_* was set on the left side with blue, while which corresponding to *z_b_* was set on the right with red. The attitude angles and control inputs are also given, from which it can be seen that the roll angle of the aerial platform will vibrate when the contact occurs, and will tend to be stable after a few seconds. The results show that the proposed controller could achieve high-precision tracking and stable physical interaction of the end effector, which verified the effectiveness of the designed controller.

## 5. Conclusions

An innovative aerial manipulator with tandem ducted fans was introduced in this paper. Compared with traditional UAV platforms, such as helicopters and multirotors, the proposed aerial manipulator could interact with the complex confined environment more closely and operate from side-on easily with a smaller range of joint motion. Considering the challenges during the manipulation process, a composite controller was proposed. An adaptive auxiliary controller was proposed for the aerial platform to compensate for the disturbances acting on the platform from the arm. The experiment results show that the designed adaptive controller had better performance than the basic controller, and verified the good stability and positioning performance of the aerial manipulator under the manipulator motion. An impedance controller was designed for the manipulator, and the experiment results show that the designed controller could ensure compliance and stability during physical interaction process. Future work will focus on the GPS-based outdoor tests, and more practical manipulation tests such as grasping will be performed and studied.

## Figures and Tables

**Table 1:** Structural parameters of the aerial manipulator.

Parameter	Physical Description	Value
*m*_total_	Total mass of the aerial manipulator	5.5 kg
*m*_b_	Mass of the vehicle platform	4.6 kg
*I*_xx_	Inertia tensor of the vehicle around x-axis	0.092 kg·m^2^
*I*_yy_	Inertia tensor of the vehicle around y-axis	0.283 kg·m^2^
*I*_zz_	Inertia tensor of the vehicle around z-axis	0.245 kg·m^2^
*p*_cd_	Distance between duct center and C.G. of vehicle	0.32 m
*R*	Duct radius	0.165 m
*n*	Blade number of each disc	4
*c*	Blade chord length	0.027 m
*θ*_0_	Attack angle at the root of blade	35 deg

**Table 2:** D–H parameters of the manipulator.

Link	a (m)	α (deg)	d (m)	θ Range (deg)
**1**	0	90	0.08	180 [−90, 90]
**2**	0.15	0	0	150 [0, 150]
**3**	0.16	0	0	150 [−150, 0]

**Table 3:** Root mean square error (RMSE) of Scenario 1.

State Variables	*x_b_* (m)	*y_b_* (m)	*z_b_* (m)	*φ* (deg)	*θ* (deg)	*ψ* (deg)
**Basic Controller**	0.0320	0.1305	0.0272	1.6484	0.7839	2.8236
**Adaptive Controller**	0.0176	0.0252	0.0126	1.0476	0.6448	0.6304

**Table 4:** RMSE of Scenario 2.

State Variables	*x_b_* (m)	*y_b_* (m)	*z_b_* (m)	*φ* (deg)	*θ* (deg)	*ψ* (deg)
**Basic Controller**	0.2324	0.2944	0.0399	1.9523	1.3520	2.8339
**Adaptive Controller**	0.1837	0.1735	0.0187	1.9606	1.7468	0.5584
